# Supplementary material for: A Likelihood Approach for Real-Time Calibration of Stochastic Compartmental Epidemic Models
Source: PLoS Comput Biol. 2017 Jan 17;13(1):e1005257. doi: 10.1371/journal.pcbi.1005257 (PMC5240920; doi:10.1371/journal.pcbi.1005257)
Supplement: S1 File — (TAR.GZ) [file pcbi.1005257.s014.tar.gz › HSPH_Online-SI-Revision/PF08/n10-extreme/n10-extreme_PF2_table-8.pdf]

1	newly 3 weeks spec	116.	248.38	{51., 613.}	148.138	0.355871	newly 3 weeks cumul	174.	525.81	{129., 1210.}	212.868	0.390479	newly 5 weeks spec	547.	405.5	{67., 1036.}	65.0311	0.449189	newly 5 weeks cumul	988.	1267.44	{254., 3045.}	86.1134	0.342722	newly 66 weeks cumul	9069.	6770.31	{3426., 8870.}	25.711	0.152539
2	newly 3 weeks spec	211.	258.36	{152., 397.}	35.9526	0.127079	newly 3 weeks cumul	450.	629.08	{397., 937.}	45.4622	0.146944	newly 5 weeks spec	408.	353.85	{196., 517.}	30.2868	0.150936	newly 5 weeks cumul	1168.	1294.51	{762., 1964.}	30.8142	0.119941	newly 62 weeks cumul	7262.	6867.77	{5626., 7972.}	10.1293	0.0476541
3	newly 3 weeks spec	63.	297.43	{16., 705.}	404.714	0.629349	newly 3 weeks cumul	84.	578.61	{40., 1383.}	609.393	0.705611	newly 5 weeks spec	540.	508.	{23., 1245.}	78.3593	0.554124	newly 5 weeks cumul	830.	1509.58	{111., 3734.}	138.111	0.495729	newly 68 weeks cumul	9108.	6690.45	{2433., 8880.}	26.7131	0.200133
4	newly 3 weeks spec	319.	249.58	{142., 386.}	33.2163	0.177355	newly 3 weeks cumul	641.	622.1	{384., 873.}	27.5694	0.123008	newly 5 weeks spec	626.	335.78	{190., 541.}	48.3099	0.313389	newly 5 weeks cumul	1754.	1252.24	{773., 1881.}	35.862	0.201246	newly 62 weeks cumul	8332.	6561.66	{5330., 7948.}	21.4388	0.108885
5	newly 3 weeks spec	240.	265.58	{78., 593.}	67.5333	0.290614	newly 3 weeks cumul	440.	585.02	{204., 1246.}	71.1955	0.246483	newly 5 weeks spec	689.	410.61	{117., 969.}	56.4456	0.413498	newly 5 weeks cumul	1573.	1339.4	{423., 3014.}	56.6408	0.311128	newly 65 weeks cumul	9428.	7190.07	{5074., 9124.}	23.7371	0.127451
6	newly 3 weeks spec	128.	269.3	{54., 633.}	141.688	0.348219	newly 3 weeks cumul	239.	572.37	{137., 1272.}	156.933	0.338366	newly 5 weeks spec	489.	428.85	{83., 901.}	63.2699	0.378117	newly 5 weeks cumul	1036.	1358.96	{289., 3005.}	82.6139	0.318284	newly 66 weeks cumul	9287.	6922.87	{3561., 8848.}	25.4718	0.150637
7	newly 3 weeks spec	131.	301.45	{58., 706.}	160.664	0.328372	newly 3 weeks cumul	207.	620.4	{158., 1304.}	210.56	0.356002	newly 5 weeks spec	469.	442.46	{84., 1177.}	69.1855	0.375902	newly 5 weeks cumul	924.	1446.54	{320., 3627.}	101.955	0.312681	newly 67 weeks cumul	8335.	6804.28	{3648., 9066.}	22.7638	0.132194
8	newly 3 weeks spec	187.	238.15	{93., 448.}	60.9465	0.214161	newly 3 weeks cumul	315.	555.19	{231., 1015.}	86.5365	0.229321	newly 5 weeks spec	589.	350.8	{129., 743.}	52.146	0.359393	newly 5 weeks cumul	1255.	1202.16	{447., 2378.}	51.753	0.244073	newly 64 weeks cumul	9223.	6804.33	{5228., 8498.}	26.2243	0.139063
9	newly 3 weeks spec	245.	228.08	{97., 430.}	48.5143	0.23023	newly 3 weeks cumul	451.	532.51	{255., 980.}	48.5078	0.179962	newly 5 weeks spec	556.	336.11	{121., 665.}	51.5522	0.347999	newly 5 weeks cumul	1389.	1151.3	{506., 2286.}	47.2671	0.249209	newly 64 weeks cumul	8874.	6716.86	{4932., 8627.}	24.5217	0.131362
10	newly 3 weeks spec	219.	267.7	{105., 533.}	58.5297	0.219441	newly 3 weeks cumul	414.	623.51	{257., 1188.}	69.6739	0.212146	newly 5 weeks spec	476.	388.52	{143., 757.}	45.5588	0.258669	newly 5 weeks cumul	1223.	1347.44	{514., 2568.}	51.5045	0.214752	newly 64 weeks cumul	8927.	6776.92	{5102., 8390.}	24.1924	0.127046
11	newly 3 weeks spec	260.	239.63	{181., 318.}	19.7115	0.0905347	newly 3 weeks cumul	477.	629.99	{495., 872.}	32.5304	0.113235	newly 5 weeks spec	539.	293.	{224., 382.}	45.6401	0.274135	newly 5 weeks cumul	1360.	1191.55	{935., 1580.}	20.2228	0.0960887	newly 59 weeks cumul	8770.	6091.75	{5557., 6723.}	30.5388	0.159435
12	newly 3 weeks spec	147.	223.44	{105., 424.}	65.8503	0.19613	newly 3 weeks cumul	271.	521.75	{261., 894.}	95.2288	0.250818	newly 5 weeks spec	376.	331.87	{143., 629.}	47.0718	0.23867	newly 5 weeks cumul	836.	1129.31	{517., 2100.}	55.6663	0.183267	newly 64 weeks cumul	8374.	6683.74	{4862., 8579.}	21.5001	0.11348
13	newly 3 weeks spec	356.	235.07	{157., 354.}	36.177	0.211062	newly 3 weeks cumul	666.	609.49	{434., 910.}	25.0706	0.116645	newly 5 weeks spec	681.	288.28	{189., 444.}	57.6681	0.396411	newly 5 weeks cumul	1797.	1160.15	{795., 1783.}	36.9911	0.215587	newly 60 weeks cumul	8214.	5925.13	{5011., 7093.}	27.8655	0.145273
14	newly 3 weeks spec	267.	257.96	{100., 506.}	51.6255	0.239644	newly 3 weeks cumul	466.	581.57	{238., 1025.}	54.0665	0.191016	newly 5 weeks spec	683.	402.86	{128., 822.}	51.0161	0.358092	newly 5 weeks cumul	1583.	1318.65	{488., 2526.}	48.2167	0.258766	newly 65 weeks cumul	8671.	7053.33	{4880., 8769.}	19.7399	0.104017
15	newly 3 weeks spec	284.	233.75	{132., 360.}	33.8627	0.176312	newly 3 weeks cumul	552.	586.62	{362., 868.}	31.3406	0.12766	newly 5 weeks spec	600.	317.22	{174., 535.}	48.51	0.321164	newly 5 weeks cumul	1572.	1181.96	{682., 1890.}	35.2188	0.193785	newly 62 weeks cumul	7388.	6358.72	{5205., 7581.}	15.542	0.0765075
16	newly 3 weeks spec	140.	270.73	{85., 461.}	111.779	0.288507	newly 3 weeks cumul	241.	598.66	{205., 1012.}	154.573	0.339957	newly 5 weeks spec	517.	424.23	{120., 787.}	49.2205	0.294773	newly 5 weeks cumul	1029.	1373.52	{422., 2289.}	68.3207	0.246948	newly 65 weeks cumul	8875.	7207.33	{5422., 8939.}	19.5634	0.101358
17	newly 3 weeks spec	117.	433.45	{49., 933.}	292.128	0.484674	newly 3 weeks cumul	203.	846.7	{121., 1835.}	330.433	0.510023	newly 5 weeks spec	499.	633.68	{91., 1360.}	85.1623	0.393552	newly 5 weeks cumul	983.	2045.9	{266., 4214.}	145.864	0.397586	newly 67 weeks cumul	8786.	7419.76	{4874., 9273.}	18.2766	0.0976838
18	newly 3 weeks spec	167.	271.08	{90., 549.}	92.4671	0.270646	newly 3 weeks cumul	309.	609.79	{222., 1162.}	110.107	0.270343	newly 5 weeks spec	657.	421.72	{124., 869.}	51.449	0.361053	newly 5 weeks cumul	1340.	1384.56	{461., 2791.}	58.8672	0.269693	newly 65 weeks cumul	9346.	7078.74	{5054., 9022.}	24.282	0.130588
19	newly 3 weeks spec	61.	354.28	{48., 816.}	489.41	0.590944	newly 3 weeks cumul	87.	718.45	{123., 1627.}	727.391	0.738166	newly 5 weeks spec	390.	525.68	{75., 1212.}	87.0718	0.354924	newly 5 weeks cumul	643.	1702.52	{268., 3823.}	188.753	0.399139	newly 67 weeks cumul	8626.	7178.17	{4824., 9008.}	18.4622	0.099518
20	newly 3 weeks spec	181.	206.01	{99., 396.}	49.895	0.19306	newly 3 weeks cumul	336.	488.46	{252., 866.}	58.4762	0.176967	newly 5 weeks spec	555.	308.8	{138., 692.}	53.0595	0.366029	newly 5 weeks cumul	1224.	1056.7	{497., 2073.}	45.5605	0.229558	newly 64 weeks cumul	9145.	6625.15	{4918., 8525.}	27.5544	0.147614
21	newly 3 weeks spec	233.	226.31	{145., 306.}	24.9914	0.112259	newly 3 weeks cumul	468.	576.74	{385., 764.}	30.4359	0.109131	newly 5 weeks spec	546.	300.84	{192., 444.}	45.4286	0.285647	newly 5 weeks cumul	1440.	1141.98	{752., 1528.}	27.3056	0.144431	newly 61 weeks cumul	8562.	6378.17	{5366., 7357.}	25.5061	0.131468
22	newly 3 weeks spec	42.	291.73	{16., 705.}	619.262	0.713188	newly 3 weeks cumul	65.	567.5	{40., 1266.}	789.538	0.771458	newly 5 weeks spec	339.	499.58	{23., 1245.}	111.499	0.513478	newly 5 weeks cumul	532.	1481.86	{111., 3734.}	219.62	0.522932	newly 68 weeks cumul	9331.	6641.15	{2433., 8880.}	28.8401	0.213626
23	newly 3 weeks spec	289.	235.47	{102., 454.}	44.4533	0.238984	newly 3 weeks cumul	567.	561.84	{261., 990.}	41.2945	0.18494	newly 5 weeks spec	502.	332.82	{135., 667.}	48.0319	0.299779	newly 5 weeks cumul	1436.	1180.85	{528., 2352.}	43.7249	0.231666	newly 63 weeks cumul	6906.	6615.7	{4867., 8298.}	15.4509	0.0719195
24	newly 3 weeks spec	396.	255.67	{151., 344.}	38.1035	0.223462	newly 3 weeks cumul	794.	652.18	{403., 856.}	25.8136	0.13415	newly 5 weeks spec	721.	327.69	{201., 478.}	54.7975	0.369202	newly 5 weeks cumul	2068.	1274.94	{764., 1716.}	40.1083	0.238598	newly 61 weeks cumul	8926.	6233.42	{5113., 7424.}	30.1656	0.160684
25	newly 3 weeks spec	77.	210.75	{89., 403.}	175.545	0.366189	newly 3 weeks cumul	116.	492.61	{244., 867.}	324.664	0.568111	newly 5 weeks spec	441.	319.06	{121., 781.}	53.424	0.31544	newly 5 weeks cumul	781.	1079.46	{480., 2215.}	62.4994	0.198063	newly 64 weeks cumul	9000.	6581.8	{4856., 8476.}	27.0282	0.146122
26	newly 3 weeks spec	130.	396.06	{54., 885.}	229.677	0.420494	newly 3 weeks cumul	214.	800.84	{137., 1739.}	287.458	0.459038	newly 5 weeks spec	577.	558.58	{76., 1204.}	69.4385	0.397426	newly 5 weeks cumul	1076.	1864.82	{282., 4133.}	115.216	0.350117	newly 67 weeks cumul	7335.	7181.59	{4133., 9219.}	20.5667	0.102136
27	newly 3 weeks spec	133.	264.86	{78., 437.}	115.203	0.302268	newly 3 weeks cumul	225.	578.7	{209., 932.}	160.827	0.358156	newly 5 weeks spec	482.	425.41	{117., 728.}	45.417	0.261586	newly 5 weeks cumul	995.	1352.72	{434., 2316.}	66.3216	0.239802	newly 65 weeks cumul	9083.	7294.65	{5511., 9107.}	19.9843	0.104166
28	newly 3 weeks spec	69.	300.47	{17., 765.}	368.681	0.603188	newly 3 weeks cumul	105.	629.79	{41., 1398.}	522.867	0.651854	newly 5 weeks spec	530.	477.86	{29., 1245.}	78.2679	0.554768	newly 5 weeks cumul	861.	1504.72	{116., 3798.}	132.065	0.482822	newly 68 weeks cumul	9312.	6493.17	{2360., 8979.}	30.2881	0.227126
29	newly 3 weeks spec	156.	199.61	{106., 356.}	48.1731	0.158293	newly 3 weeks cumul	228.	481.	{288., 789.}	111.175	0.283449	newly 5 weeks spec	612.	284.26	{128., 516.}	56.9673	0.413682	newly 5 weeks cumul	1151.	1006.25	{539., 1749.}	41.8949	0.203023	newly 63 weeks cumul	9014.	6502.36	{5140., 7980.}	27.8638	0.148134
30	newly 3 weeks spec	209.	222.71	{109., 364.}	42.1196	0.173394	newly 3 weeks cumul	375.	537.03	{270., 854.}	55.4053	0.172592	newly 5 weeks spec	521.	310.32	{130., 565.}	48.7678	0.318307	newly 5 weeks cumul	1271.	1118.18	{524., 1854.}	40.8356	0.203139	newly 63 weeks cumul	8143.	6419.66	{4652., 7873.}	22.0533	0.11568
31	newly 3 weeks spec	173.	289.5	{64., 585.}	104.347	0.321349	newly 3 weeks cumul	303.	620.35	{142., 1226.}	126.01	0.318705	newly 5 weeks spec	607.	452.55	{88., 952.}	54.1829	0.371757	newly 5 weeks cumul	1297.	1453.53	{334., 2959.}	66.5127	0.305204	newly 66 weeks cumul	9186.	6749.06	{3413., 8854.}	26.6786	0.157072
32	newly 3 weeks spec	258.	249.51	{184., 320.}	17.2597	0.0772236	newly 3 weeks cumul	565.	663.59	{517., 847.}	21.2195	0.0792707	newly 5 weeks spec	577.	296.4	{220., 376.}	48.6308	0.298791	newly 5 weeks cumul	1557.	1233.79	{958., 1591.}	23.1683	0.118358	newly 58 weeks cumul	8222.	5871.8	{5347., 6496.}	28.5843	0.14755
33	newly 3 weeks spec	126.	273.21	{47., 606.}	145.579	0.351356	newly 3 weeks cumul	184.	571.17	{131., 1205.}	220.864	0.414749	newly 5 weeks spec	733.	458.09	{72., 1158.}	59.382	0.457159	newly 5 weeks cumul	1177.	1401.27	{254., 3227.}	75.0068	0.323399	newly 66 weeks cumul	9187.	6864.34	{3587., 9270.}	25.6567	0.152091
34	newly 3 weeks spec	135.	302.29	{16., 695.}	177.77	0.540815	newly 3 weeks cumul	190.	685.58	{40., 1357.}	297.884	0.541813	newly 5 weeks spec	568.	462.14	{23., 1184.}	76.3451	0.615744	newly 5 weeks cumul	1068.	1531.21	{111., 3652.}	110.765	0.505952	newly 68 weeks cumul	8375.	6286.1	{1393., 8873.}	27.4543	0.241116
35	newly 3 weeks spec	297.	234.04	{113., 387.}	39.569	0.215432	newly 3 weeks cumul	580.	571.	{299., 938.}	34.5586	0.154485	newly 5 weeks spec	563.	330.31	{166., 589.}	47.2735	0.306503	newly 5 weeks cumul	1559.	1179.25	{607., 2089.}	39.9096	0.220842	newly 63 weeks cumul	8157.	6244.99	{4473., 8034.}	24.2757	0.130049
36	newly 3 weeks spec	233.	223.92	{141., 335.}	27.2961	0.124782	newly 3 weeks cumul	424.	565.96	{373., 806.}	38.4151	0.130806	newly 5 weeks spec	562.	288.8	{177., 421.}	48.6121	0.312793	newly 5 weeks cumul	1395.	1112.55	{729., 1611.}	27.8631	0.147589	newly 61 weeks cumul	9215.	6377.71	{5394., 7309.}	30.7899	0.162574
37	newly 3 weeks spec	197.	211.12	{96., 392.}	48.6294	0.204967	newly 3 weeks cumul	298.	490.06	{238., 860.}	73.3423	0.2082	newly 5 weeks spec	605.	321.21	{112., 661.}	52.6198	0.380947	newly 5 weeks cumul	1277.	1078.91	{457., 2011.}	44.1135	0.233787	newly 64 weeks cumul	9224.	6720.25	{4963., 8412.}	27.1439	0.146121
38	newly 3 weeks spec	40.	337.52	{48., 791.}	747.1	0.71746	newly 3 weeks cumul	62.	688.28	{123., 1560.}	1010.74	0.857393	newly 5 weeks spec	491.	509.23	{70., 1204.}	71.057	0.379642	newly 5 weeks cumul	699.	1637.34	{268., 3783.}	163.765	0.382305	newly 67 weeks cumul	8849.	7179.48	{4769., 9008.}	19.6908	0.106948
39	newly 3 weeks spec	103.	375.35	{55., 733.}	280.34	0.453241	newly 3 weeks cumul	146.	749.04	{159., 1534.}	418.014	0.563234	newly 5 weeks spec	645.	571.12	{89., 1254.}	61.262	0.383647	newly 5 weeks cumul	1060.	1817.38	{327., 3858.}	109.796	0.337898	newly 67 weeks cumul	9368.	7153.69	{3860., 9400.}	23.8406	0.139521
40	newly 3 weeks spec	276.	255.85	{104., 478.}	44.5688	0.218886	newly 3 weeks cumul	519.	597.14	{268., 1067.}	45.5723	0.179622	newly 5 weeks spec	547.	384.22	{141., 787.}	47.4406	0.296815	newly 5 weeks cumul	1464.	1304.26	{519., 2470.}	43.4249	0.221949	newly 64 weeks cumul	8627.	6848.02	{4937., 8849.}	21.6154	0.114885
41	newly 3 weeks spec	249.	198.31	{103., 347.}	42.0361	0.221674	newly 3 weeks cumul	416.	486.87	{278., 787.}	40.1466	0.145152	newly 5 weeks spec	680.	274.26	{137., 536.}	60.3676	0.456909	newly 5 weeks cumul	1527.	997.39	{538., 1806.}	45.6392	0.272315	newly 63 weeks cumul	9091.	6271.99	{4879., 7392.}	31.0088	0.167873
42	newly 3 weeks spec	386.	252.63	{202., 314.}	34.5674	0.190512	newly 3 weeks cumul	794.	674.06	{559., 810.}	17.1839	0.084072	newly 5 weeks spec	673.	294.96	{232., 356.}	56.1724	0.36456	newly 5 weeks cumul	2037.	1242.14	{1021., 1503.}	39.0211	0.219568	newly 58 weeks cumul	8720.	5743.26	{5218., 6346.}	34.1369	0.182973
43	newly 3 weeks spec	32.	297.43	{16., 705.}	849.844	0.793766	newly 3 weeks cumul	43.	578.61	{40., 1383.}	1255.98	0.907274	newly 5 weeks spec	315.	508.	{23., 1245.}	122.597	0.518702	newly 5 weeks cumul	461.	1509.58	{111., 3734.}	264.512	0.550019	newly 68 weeks cumul	9060.	6690.45	{2433., 8880.}	26.3983	0.198156
44	newly 3 weeks spec	255.	251.25	{183., 357.}	19.8235	0.0859905	newly 3 weeks cumul	527.	652.19	{507., 918.}	25.444	0.0899119	newly 5 weeks spec	597.	303.86	{223., 434.}	49.1022	0.305951	newly 5 weeks cumul	1543.	1232.58	{944., 1736.}	25.1899	0.127996	newly 59 weeks cumul	8693.	6160.12	{5597., 6864.}	29.137	0.151057
45	newly 3 weeks spec	293.	203.05	{100., 327.}	44.7747	0.257296	newly 3 weeks cumul	562.	497.03	{268., 795.}	36.7242	0.174736	newly 5 weeks spec	606.	283.64	{131., 446.}	57.1122	0.404009	newly 5 weeks cumul	1576.	1025.64	{531., 1619.}	45.6358	0.271417	newly 63 weeks cumul	8765.	6323.44	{4615., 7531.}	27.9537	0.149674
46	newly 3 weeks spec	273.	248.28	{146., 371.}	30.6374	0.145978	newly 3 weeks cumul	518.	624.45	{373., 906.}	34.0637	0.123569	newly 5 weeks spec	572.	322.3	{201., 497.}	45.2028	0.285494	newly 5 weeks cumul	1495.	1234.69	{755., 1861.}	30.2415	0.156244	newly 61 weeks cumul	8858.	6371.14	{5362., 7357.}	28.0747	0.146346
47	newly 3 weeks spec	140.	199.82	{97., 356.}	58.3286	0.180977	newly 3 weeks cumul	227.	490.63	{265., 857.}	117.229	0.294805	newly 5 weeks spec	505.	280.99	{122., 491.}	51.9782	0.344133	newly 5 weeks cumul	1027.	1014.81	{509., 1767.}	39.6699	0.174332	newly 63 weeks cumul	9302.	6184.7	{4603., 7544.}	33.5121	0.185746
48	newly 3 weeks spec	115.	276.79	{47., 641.}	165.783	0.365439	newly 3 weeks cumul	204.	583.91	{133., 1265.}	198.426	0.386901	newly 5 weeks spec	483.	450.42	{72., 1047.}	67.7019	0.387553	newly 5 weeks cumul	959.	1404.52	{260., 3124.}	93.2555	0.325965	newly 66 weeks cumul	9126.	6756.38	{3413., 9109.}	26.304	0.155947
49	newly 3 weeks spec	85.	337.52	{48., 791.}	314.424	0.481589	newly 3 weeks cumul	133.	688.28	{123., 1560.}	423.654	0.557714	newly 5 weeks spec	380.	509.23	{70., 1204.}	87.7868	0.364571	newly 5 weeks cumul	694.	1637.34	{268., 3783.}	165.205	0.383427	newly 67 weeks cumul	8859.	7179.48	{4769., 9008.}	19.7544	0.10732
50	newly 3 weeks spec	88.	264.19	{96., 539.}	202.08	0.395594	newly 3 weeks cumul	156.	584.1	{237., 1101.}	274.423	0.4999	newly 5 weeks spec	376.	417.43	{132., 852.}	58.7101	0.254339	newly 5 weeks cumul	733.	1345.65	{474., 2692.}	98.7681	0.261476	newly 65 weeks cumul	9078.	7150.23	{4962., 9065.}	21.517	0.114129
